# Japan's Conditional/Time‐Limited Early Approval System in Regenerative Medicine: A Case Study of Rise and Falls of Autologous Skeletal Myoblast Sheets

**DOI:** 10.1002/cpt.3562

**Published:** 2025-01-14

**Authors:** Hayase Hakariya, Akihiko Ozaki, Yudai Kaneda, Tetsuya Tanimoto

**Affiliations:** ^1^ Interfaculty Institute of Biochemistry University of Tuebingen Tuebingen Germany; ^2^ Institute for Pharmaceutical and Social Health Sciences Ise Japan; ^3^ Department of Breast and Thyroid Surgery Jyoban Hospital of Tokiwa Foundation Iwaki Fukushima Japan; ^4^ School of Medicine Hokkaido University Sapporo Hokkaido Japan; ^5^ Navitas Clinic Tokyo Japan

## Abstract

Japan's conditional/time‐limited early approval program, initiated in 2014, aimed to advance regenerative medicine by expediting market access. However, the withdrawal of autologous skeletal myoblast sheets (Heartsheet) due to ineffectiveness raises concerns about the balance between rapid approval and scientific integrity. While the program seeks to boost innovation, it risks endorsing costly, unclear treatments under national health care. This case highlights the need to refine regulatory approaches, ensuring clinical efficacy and fiscal responsibility in regenerative therapies.

Japan introduced the conditional/time‐limited early approval (CEA) program, which is similar to the accelerated approval pathway in the United States and was introduced in Japan in 2014, specifically in the field of regenerative medicine to promote innovative drug development.^1^ The program expanded the scope to include medical drugs other than products for regenerative medicine in October 2017. The program became legislative under the revised Pharmaceuticals and Medical Devices Act in November 2019.^2^ Among a total of nine products granted the CEA as of November 2024, four (44.4%) are classified as *New Regenerative Medicine Products*.

Currently, the Japanese government authorizes a total of 21 *New Regenerative Medical Products*, including 17 regenerative medical products and four gene therapy products (**Table** **1**). Among the 17 regenerative medical products, two (11.8%) were approved under the CEA, and the autologous skeletal myoblast sheets (Heartsheet) is the only product that has been withdrawn from the market due to the failure to provide efficacy evidence in the post‐marketing trial. Another regenerative medical product STEMIRAC developed by Nipro Cooperation, a human autologous bone marrow‐derived mesenchymal stem cell for the treatment of spinal cord injury, is still under the timeframe of the CEA (**Table** **2**). Among four gene therapy products, two (50%) were approved under the CEA and one has been withdrawn by the sponsor again due to the failure to provide efficacy evidence in the post‐marketing trial, the Collategene developed by Mitsubishi Tanabe Pharma Corporation and AnGes, Inc. for the treatment of critical limb ischemia (**Tables** **1** **and** **2**). We summarized four clinical trial results that were submitted to apply the market authorization through the CEA pathway among *New Regenerative Medical Products*, which are available from Japan's Pharmaceuticals and Medical Devices Agency review report (**Table** **2**). In most cases, their conditional approval was seemingly reasonable to some extent within the limited evidence provided. In contrast, it was noteworthy that the HeartSheet was the only product approved without any improvement reported at the time of the indication application. Herein, we trace back the case of this product to review the future direction of the CEA program.

**Table 1:** List of regenerative medical products approved in Japan^a^

No.	Brand name	Generic name	Indication	Regulatory status	Date of approval
Regenerative medical products					
1	JACE	Human (Autologous) Epidermis‐derived Cell Sheet	Serious and extensive burns Giant congenital melanocytic nevus DEB and JEB	Regular Approval	8/6/2007
2	JACC	Human (Autologous) cartilage‐derived Cell Sheet	Knee cartilage defect	Regular Approval	7/27/2012
3	TEMCELL	Human (allogeneic) bone marrow‐derived mesenchymal stem cell	Acute GVHD	Regular Approval	9/18/2015
4	HeartSheet	Human (autologous) skeletal myoblast‐derived cell sheet	Serious heart failure caused by ischemic heart disease	CEA>Withdrawal	9/18/2015
5	STEMIRAC	Human (autologous) bone marrow‐derived mesenchymal stem cell	Spinal cord injury	CEA	12/28/2018
6	Kymriah	Tisagenlecleucel	CD19‐positive relapsed or refractory B‐cell ALL CD19‐positive relapsed or refractory diffuse LBCL	Regular Approval	3/26/2019
7	Nepic	Human (autologous) corneal limbus‐derived corneal epithelial cell sheet	LSCD	Regular Approval	3/19/2020
8	YESCARTA	Axicabtagene ciloleucel	Relapsed or refractory LBCL	Regular Approval	1/22/2021
9	Breyanzi	Lisocabtagene maraleucel	Relapsed or refractory LBCL Relapsed or refractory FL	Regular Approval	3/22/2021
10	Ocural	Human (autologous) oral mucosa‐derived epithelial cell sheet	LSCD	Regular Approval	6/11/2021
11	Alofisel	Darvadstrocel	Crohn's disease	Regular Approval	9/27/2021
12	Sakracy	Human (autologous) oral mucosa‐derived epithelial cell sheet using a human amniotic membrane substrate	LSCD	Regular Approval	1/20/2022
13	Abecma	Idecabtagene vicleucel	Relapsed or refractory multiple myeloma	Regular Approval	1/20/2022
14	Carvykti	Ciltacabtagene autoleucel	Relapsed or refractory multiple myeloma	Regular Approval	9/26/2022
15	JACEMIN	Melanocytecontaining Human (Autologous) Epidermis‐derived Cell Sheet	Vitiligo	Regular Approval	3/17/2022
16	Vyznova	Neltependocel	Bullous keratopathy	Regular Approval	3/17/2022
17	AKUUGO	Vandefitemcel	Traumatic brain injury	Regular Approval	7/31/2024
Gene therapy products					
1	Collategene	Beperminogene perplasmid	Arteriosclerosis obliterans and Burger's disease	CEA > Withdrawal	3/26/2019
2	ZOLGENSMA	Onasemnogene abeparvovec	Spinal muscular atrophy	Regular Approval	3/19/2020
3	Delytact	Teserpaturev	Malignant glioma	CEA	6/11/2021
4	Luxturna	Voretigene neparvovec	inherited retinal dystrophy	Regular Approval	6/26/2023

ALL, acute lymphoblastic leukemia; CEA, conditional early approval; DEB, dystrophic epidermolysis bullosa; FL, follicular lymphoma; GVHD, graft‐versus‐host disease; JEB, junctional epidermolysis bullosa; LBCL, large B‐cell lymphoma; LCSD, limbal stem cell deficiency.

^a^ In Japan, Regenerative medical products are categorized as “New Regenerative Medical Products”, which consist of regenerative medical products and gene therapy products. As of November 2024.

**Table 2:** Major clinical trial information reviewed by the PMDA that resulted in granting approval under the conditional early approval program

Brand name	Generic name	Indication	Major clinical trials reviewed	Number of participants (FAS)	Phase	Type of study	Primary Endpoint and outcome	Regulatory status
Regenerative medical products								
HeartSheet	Human (autologous) skeletal myoblast‐derived cell sheet	Serious heart failure caused by ischemic heart disease	M‐51073‐21, multicenter (Japan)	7 (7)	2	Open‐label, uncontrolled	Change in LVEF at 26 weeks after transplantation^a^: worsened in 2, no change in 5, improved in 0 cases	CEA > Withdrawal
STEMIRAC	Human (autologous) bone marrow‐derived mesenchymal stem cell	Spinal cord injury	STR‐01‐03, single‐center (Japan)	17 (13)	2	Open‐label, uncontrolled	Proportion of cases in which the AIS score improved by one or more stages from before administration (with 7 days) on the 220 (±14) day after spinal cord injury: 92.3% (12/13)	CEA
Gene therapy products								
Collategene	Beperminogene perplasmid	ASO and Burger's disease	ASO PhaseIII^b^ multicenter (Japan)	46 (40)	3	Double‐blind, randomized	Patients with ASO (Fontaine Stage III): Improvement of VAS: 50% (8/16) in the treatment group and 25% (2/8) in the placebo group Patients with ASO (Fontaine Stage IV): Improvement of maximum ulcer size: 100% (11/11) in the treatment group and 40% (2/5) in the placebo group	CEA > Withdrawal
			TAO open‐label clinical study, multicenter (Japan)	10 (9)	2	Open‐label, uncontrolled	Improvement of maximum ulcer size among patients with Burger's disease (Fontaine Stage IV): 66.7% (6/9)	
Delytact	Teserpaturev	Malignant glioma	GD01, single‐center (Japan)	19 (16)	2	Open‐label, uncontrolled	Improvement of 1‐year survival: 92.3% (12/13)^c^	CEA

ASO, arteriosclerosis obliterans; FAS, full analysis set; VAS, visual analogue scale.

^a^ Retrospectively evaluated as ad hoc.

^b^ The sponsor submitted a further seven clinical trial data, including international studies and those performed in the United States, at the time of indication application.

^c^ An interim analysis was performed to assess early termination due to the ineffectiveness or sufficient efficacy when 13 cases completed 1 year of observation. As a result, the independent data monitoring committee recommended early termination due to the efficacy.

On July 19, 2024, Japan's Ministry of Health, Labour, and Welfare (MHLW) announced that the HeartSheet,^2^ did not accomplish their criteria for regular approval. The product was approved for the first time in 2015 through Japan's CEA pathway. In response to the MHLW's decision, the manufacturer of the HeartSheet decided to cease sales immediately on the following day. As of March 2024, the product has not granted approval nor sold in any of the countries or regions (see Supplemental Reference 1).

HeartSheet, the first global cellular and tissue‐based regenerative product prepared from autologous skeletal muscle, was originally developed by Terumo Corporation (Tokyo, Japan) and Osaka University, and designed for treating patients with severe heart failure caused by ischemic heart disease (New York Heart Association (NYHA) classification III or IV, and left ventricular ejection fraction (LVEF) at rest of 35% or less), for which standard treatments, such as drug therapy and invasive therapy are ineffective.^3^

The product's CEA in 2015 was based on a non‐randomized domestic clinical trial involving only seven patients.^4^ This preliminary study lacked a primary efficacy endpoint; This was a simple case series study in which the change in LVEF at 26 weeks after transplantation was retrospectively evaluated as ad hoc. Though no serious arrhythmia was reported, the efficacy was inconclusive as the LVEF was unchanged in five out of seven patients, and worsened in the other two.^4^, ^5^ Nevertheless, the Japanese government granted the CEA to the indication, citing that its efficacy could be expected.^6^ The authorization indicated that the therapy was covered by national universal health insurance. The government required the sponsor to perform a post‐marketing trial to verify its efficacy by measuring the survival period within a period of 5 years under CEA, which was extended for 3 years due to the lack of sample size. As a result of the post‐market study, the sponsor applied for the regular approval of HeartSheet with a dataset that compared 49 treated patients and 102 patients in the control group in 2024 (see Supplemental Reference 1). However, the MHLW committee concluded that the product is not eligible for regular approval, as they failed to demonstrate the primary endpoint (the extension of the period until cardiovascular‐related death) nor the secondary endpoint (the improvement of patients' LVEF). In this sense, the Japanese regulatory body responded to the submitted application for regular approval in a scientifically fair manner, indicating that the CEA pathway functioned appropriately.

However, it is questionable if the product was worthy of being granted the CEA originally. Japan initially established the CEA program under the economic growth policy so that the country could globally lead regenerative medicine, including cell therapy based on induced pluripotent stem cells (iPSCs). While it is imperative for Japan to develop domestic products given their stagnant growth in the pharmaceutical industry (see Supplementary Reference 2), one should also consider the balance between scientific integrity, as patients may receive ineffective or potentially unsafe treatments with therapies that have uncertain efficacy and safety.^5^, ^7^, ^8^

Moreover, covering costs for such scientifically uncertain products with the national healthcare budget should also be questioned. Costs for emerging regenerative medicine and cell therapies are generally expensive: that is, costs for HeartSheet per treatment are 14.76 million JPY (approximately 99 thousand USD) (see Supplemental Reference 3).

Products similar to the HeartSheet for the treatment of severe ischemic heart diseases have been developed by other Japanese biomedical startups, such as Cuorips Inc. and Heartseed Inc., both of which have iPSC‐derived regenerative medicine products in their pipeline. Although the clinical benefits of these products have yet to be evaluated, Japan may potentially experience similar consequences of HeartSheet due to the CEA framework; Japan may risk patients receiving treatments with regenerative medicine products that lack clinical benefits and suffer from swelled national healthcare budgets with unsuccessful therapies for years. Indeed, the MHLW's subcommittee agreed on the direction of revising the CEA system in preparation for the next revision of the Pharmaceutical and Medical Device Act, where they allow the earlier indication application than the current system by introducing the withdrawal regulation if the approved product failed to provide efficacy and safety evidence in the post‐marketing clinical trials. We underscore that Japan should carefully reform the CEA pathway, that is, by elaborating further criteria for the approval to minimize the time that drugs with uncertain clinical benefits are allowed to remain on the market. This includes the initiation of confirmatory trials before the CEA and proactively outlining plans for CEA subsequent clinical benefit verification. Such measures were taken in the case of the United States’ accelerated approvals.^9^, ^10^

## FUNDING

No funding was received for this work.

## CONFLICT OF INTEREST

T.T. receives personal fees from Medical Network Systems MNES Inc., and Bionics Inc., outside the submitted work. A.O. receives personal fees from MNES Inc., Becton, Dickinson and Company, Taiho Pharmaceutical Co., Ltd., and Kyowa Kirin Inc., outside the submitted work.

## Supporting information


Data S1
