# Supplementary material for: Japan's Conditional/Time‐Limited Early Approval System in Regenerative Medicine: A Case Study of Rise and Falls of Autologous Skeletal Myoblast Sheets
Source: Clin Pharmacol Ther. 2025 Jan 14;117(5):1171–4. doi: 10.1002/cpt.3562 (PMC11993280; doi:10.1002/cpt.3562)
Supplement: Supplementary file 1 — Data S1 [file CPT-117-1171-s001.docx]

**Supplemental Material**

**Japan’s conditional/time-limited early approval system in regenerative medicine: A case study of rise and falls of autologous skeletal myoblast sheets**

**Hayase Hakariya^1,2*^, Akihiko Ozaki^3^, Yudai Kaneda^4^, Tetsuya Tanimoto^5^**

*1; Interfaculty Institute of Biochemistry, University of Tuebingen, Tuebingen 72076, Germany*

*2; Institute for Pharmaceutical and Social Health Sciences, Japan*

*3; Department of Breast and Thyroid Surgery, Jyoban Hospital of Tokiwa Foundation, Iwaki, Fukushima 972-8322, Japan*

*4;* *School of Medicine, Hokkaido University, Hokkaido, Japan*

*5; Navitas Clinic, Tokyo, 190-0023, Japan*

ORCID, e-mail:

Hayase Hakariya : orcid.org/0000-0002-9121-4551, [hayase.hakariya@uni-tuebingen.de](mailto:hayase.hakariya@uni-tuebingen.de)
Akihiko Ozaki : orcid.org/0000-0003-4415-9657, [ozakiakihiko@gmail.com](mailto:ozakiakihiko@gmail.com)

Yudai Kaneda : orcid.org/0000-0001-8302-9439, [nature271828@gmail.com](mailto:nature271828@gmail.com)

Tetsuya Tanimoto : orcid.org/0000-0002-9818-8587, [tetanimot@yahoo.co.jp](mailto:tetanimot@yahoo.co.jp)

*To whom correspondence may be addressed.

Hayase Hakariya, Ph.D.
Interfaculty Institute of Biochemistry, University of Tuebingen, 72076 Tuebingen, Germany

TEL: +49 7071 29 75377
Email: [hayase.hakariya@uni-tuebingen.de](mailto:hayase.hakariya@uni-tuebingen.de), [haya.pha3@gmail.com](mailto:haya.pha3@gmail.com) (H.H.)

**Supplemental Contents**

[Supplemental References 2](#_Toc183673480)

## **Supplemental References**

1. Pharmaceuticals and Medical Devices Agency (PMDA), Japan. (2024) Updated review report of HeartSheet^®︎^ (manufactured by Terumo Corporation, Ltd.) [in Japanese] <https://www.pmda.go.jp/regenerative_medicines/2024/R20240808001/470034000_XXXXXXXXXXXXX_A100_1.pdf>. Accessed November 4, 2024.

2. IQVIA Institute for Human Data Science, The global use of medicines 2022: outlook to 2026. (2022) <https://www.iqvia.com/-/media/iqvia/pdfs/library/publications/the-global-use-of-medicines-2022.pdf>. Accessed August 6, 2024.

3. McCabe, C. & Sipp, D. Undertested and Overpriced: Japan Issues First Conditional Approval of Stem Cell Product. Cell Stem Cell 18, 436–437 (2016).
